# Distress and neuroticism as mediators of the effect of childhood and adulthood adversity on cognitive performance in the UK Biobank study

**DOI:** 10.1038/s41598-024-58510-z

**Published:** 2024-04-06

**Authors:** Chris Patrick Pflanz, Morgane Künzi, John Gallacher, Sarah Bauermeister

**Affiliations:** 1 Dementias Platform UK, Department of Psychiatry, Warneford Hospital, University of Oxford, Oxford, OX3 7JX UK; 2 Centre the Interdisciplinary Study of Gerontology and Vulnerability, University of Geneva, Geneva, Switzerland

**Keywords:** Human behaviour, Risk factors, Emotion, Cognitive neuroscience

## Abstract

Childhood adversity and adulthood adversity affect cognition later in life. However, the mechanism through which adversity exerts these effects on cognition remains under-researched. We aimed to investigate if the effect of adversity on cognition was mediated by distress or neuroticism. The UK Biobank is a large, population-based, cohort study designed to investigate risk factors of cognitive health. Here, data were analysed using a cross-sectional design. Structural equation models were fitted to the data with childhood adversity or adulthood adversity as independent variables, distress and neuroticism as mediators and executive function and processing speed as latent dependent variables that were derived from the cognitive scores in the UK Biobank. Complete data were available for 64,051 participants in the childhood adversity model and 63,360 participants in the adulthood adversity model. Childhood adversity did not show a direct effect on processing speed. The effect of childhood adversity on executive function was partially mediated by distress and neuroticism. The effects of adulthood adversity on executive function and processing speed were both partially mediated by distress and neuroticism. In conclusion, distress and neuroticism mediated the deleterious effect of childhood and adulthood adversity on cognition and may provide a mechanism underlying the deleterious consequences of adversity.

## Introduction

Childhood adversity is an umbrella term for adverse childhood experiences that could impair children’s health and wellbeing^1–3^. The U.S. Department of Health & Human Services distinguishes between several types of child adversity including physical abuse, psychological or emotional maltreatment, sexual abuse, sex trafficking, medical neglect, neglect or deprivation of necessities^4^. Childhood adversity can lead to lifelong physical health problems, mental health conditions and behavioural problems^5^. A large body of evidence has also linked adversity with cognitive deficits later in life^6–9^ including executive function^10^. Executive function in this context may be defined as a complex set of cognitive abilities that include working memory, inhibitory control, cognitive flexibility, planning, reasoning, and problem solving^11^. In addition to childhood adversity, the experience of adverse events during adulthood, can accumulate over the life course which is known as adulthood adversity and can have a detrimental impact on cognition^12^. However, the mechanisms underlying the effect of adversity on cognition later in life are not fully understood and are subject to further research. A potential mechanism through which adversity affects cognition is through early changes of personality during childhood that may lead to subsequent mental health conditions, including distress, and psychosocial problems. One personality trait that might be associated with early childhood adversity is neuroticism. Neuroticism may be defined as the tendency to experience negative emotions, including anxiety, depression, hostility, and mood swings^13^. Indeed, research suggests that childhood abuse increases the likelihood of developing neurotic personality traits in later life^14^.

Furthermore, previous research showed a link between neuroticism and performance in cognitive tests: Older adults high in neuroticism perceived more stress which led to lower performance in executive function tasks across a study period of six years^15^. Further research showed that in older adults, neuroticism was associated with worse performance in working memory, executive function^16^ and other cognitive performance tasks^17^. Thus, neuroticism may be a candidate mediator of the negative effect of adversity on cognitive performance because of the known association between neuroticism and adversity^14^ on one hand and the known association between neuroticism and cognition on the other hand^15–17^.

Neuroticism is also of particular interest from a theoretical perspective to further investigate the link between adversity and cognition. In the Eysenck personality model, interindividual differences along the neuroticism-emotional stability continuum are the result of interindividual differences in the neurophysiology of the limbic system that controls the reactivity of the autonomic nervous system including the sympathetic and parasympathetic branches^18^. Hyperarousal of the sympathetic nervous system has also been suggested as a contributing factor for development mental health conditions in adulthood following early childhood adversity^19^. Hyperarousal of the sympathetic nervous system is also linked to cognitive decline in old age^20^. Hence neuroticism, as marker of interindividual differences in the functioning of the autonomic nervous system may be able to further explain the three-fold association between childhood adversity, mental health and cognitive performance which is the reason why neuroticism was selected as a mediator here. Another candidate mediator is affect-related distress which is expressed as concomitant symptoms of anxiety and depression^21^. Distress in this context may be defined as non-specific symptoms of stress, anxiety and depression^22^. Anxiety and depression are known to have high comorbidity^23–25^. The DSM-5, therefore, introduced the anxious distress specifier in recognition of the clinical importance of this comorbidity in depressed patients^26^. Previous research showed that childhood adversity was associated with comorbid anxiety and depression^27^, which is the reason why the present study focused on the distress in general rather than individual symptoms of depression or anxiety.

Previous research also showed that individually anxiety and depression show high comorbidity^24,28,29^ and may be overlapping constructs of mental health-related distress^30^. Childhood adversities were also found to increase vulnerability to anxiety, depression and distress later in life as reviewed by^31^, but no association between adversity type and a specific mental health condition, e.g. depression or anxiety was found, suggesting that general vulnerability to mood and affect disorders is increased later in life. We, therefore, investigated affect-related distress as a single construct rather than considering individual types of mood disorders. We were particularly interested in affect-related distress because research showed that childhood maltreatment and trauma were associated with a greater risk of depression and anxiety disorders during adulthood^32–34^.

Depression and anxiety disorders, in turn, have been linked to deficits in cognitive function and performance^35^. Indeed, cognitive deficits often last longer than the depressive episode as so-called residual symptoms of depression^36^. In a large cohort study, cognitive and somatic depressive symptoms, measured using the PHQ-9 were associated with lower cognitive function among older adults^37^. Research also showed that older adults with depressive symptoms had lower memory, executive function, and processing speed compared to older adults without depressive symptoms and that these lower levels in cognitive performance might be explained by low brain-derived neurotrophic factor (BDNF) levels in the blood^38^. Research showed that BDNF plays a vital role in the pathophysiology of depression^39^, and that genetic BDNF polymorphisms can influence cognition^40^, thereby suggesting that low BDNF levels may provide a link between depression and cognitive performance. These findings let to the neurotrophic theory of depression that posits that decreased levels of neurotrophic factors may contribute to the atrophy of limbic brain regions (e.g. the hippocampus and prefrontal cortex) that in turn may explain symptoms of depression, and that the therapeutic actions of antidepressants may result from a reversal of this neuronal atrophy and cell loss^41^. Evidence further suggests that deficits in BDNF contribute to the pathogenesis of both depression and Alzheimer’s disease^42^. Similar to patients with depression, patients with post-traumatic stress disorder (PTSD) were found to perform poorly in cognitive tasks measuring psychomotor speed/attention and learning/working memory^43^. A meta-analysis found older adults with PTSD performed worse across a range of measures from several cognitive domains relative to older adults without PTSD^44^.

In the present study, the association between childhood or adulthood adversity and cognitive performance was investigated. Neuroticism, and affect-related distress were investigated as candidate mediators of this association. We aimed to investigate the relationship between neuroticism and affect-related distress on the effect of adversity on cognition in the UK Biobank.

## Results

### Descriptive statistics

Models were run listwise including only subjects that had complete data for the model being tested. The structural equation model on childhood adversity included 64,051 participants aged 40 to 72 years (M = 55.63 years; SD = 7.61), 54.76% females. The structural equation model on adulthood adversity included 63,360 participants aged 40 to 72 years (M = 55.62 years; SD = 7.60), 54.66% females. A frequency table for childhood and adulthood adversity is displayed in Supplementary Table S2.

### Exploratory factor analysis

The exploratory factor analysis showed that a two-dimensional factor structure describes the cognitive data in the UK Biobank best (Table 1 for factor loadings). Only two factors were retained because the eigenvalues associated with the remaining factors were negative^45^. The first factor comprised tasks measuring executive function including the Trail Making Test, the numeric memory test score, the fluid intelligence score, and the symbol digit test score. The second factor comprised a task measuring processing speed involving mean reaction time from the “snap game” and reaction time variability from the “snap game”. A correlation analysis showed that the retained factors were weakly correlated (Pearson’s r = − 0.278, *p* < 0.001).

**Table 1:** Exploratory factor analysis with an orthogonal varimax rotation on cognitive scores in the UK Biobank.

Variable	Factor 1 “Executive function”	Factor 2 “Processing speed”	Uniqueness
Correct Digits	**0.43**	**− **0.04	0.81
Fluid Intelligence	**0.53**	**− **0.10	0.71
Symbol matches	**0.41**	**− **0.23	0.78
TMTB/TMTA log ratio	**− 0.30**	0.03	0.91
Mean RT	**− **0.15	**0.49**	0.74
RT variability	**− **0.02	**0.43**	0.81
Variance	0.75	0.49	–

*N* = 64,051. Factor loadings > 0.3 are shown in bold. Only factors with positive eigenvalues were retained, yielding a two-factor solution.

*TMT* trail making test, *RT* reaction time.

### Effects of childhood adversity on cognitive performance

#### Direct effects model

**Figure 1:**
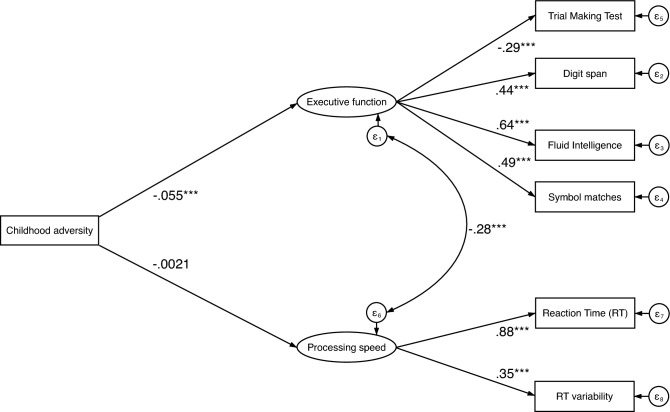
Path diagram showing the relationship between variables entered in the structural equation modelling with childhood adversity as the independent variable, executive function and processing speed as latent dependent variables. *Note:* Path-coefficients are standardized coefficients. Covariates not shown for display purposes. Covariates include sex, age, Townsend deprivation index, and education. *p < 0.05, **p < 0.01, ***p < 0.001.

A direct effects model (Fig. 1) with childhood adversity as the independent variable and executive function and processing speed as the latent, dependent variables had good model fit, CFI = 0.931, RMSEA = 0.049, and SRMR = 0.027. There was a moderate direct effect of childhood adversity on executive function (β = − 0.055, *p* < 0.001), but the direct effect of childhood adversity on processing speed was not significant (β = − 0.002, *p* = 0.621).

#### Indirect effects model

**Figure 2:**
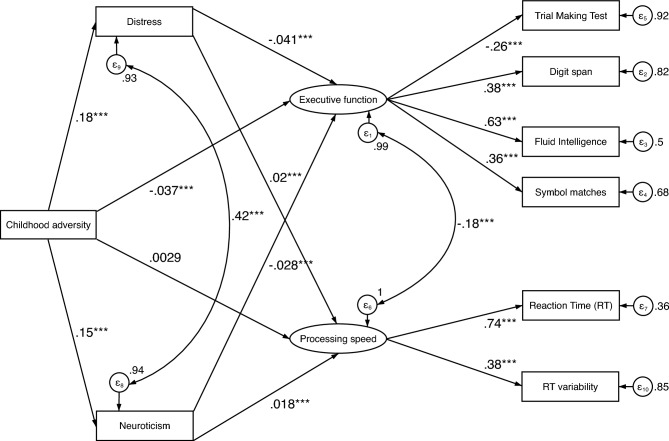
Path diagram showing the relationship between variables entered in the structural equation modelling with childhood adversity as the independent variable, executive function and processing speed as latent dependent variables and distress and neuroticism as mediators. *Note:* Path-coefficients are standardized coefficients. Covariates not shown for display purposes. Covariates include sex, age, Townsend deprivation index, and education. *p < 0.05, **p < 0.01, ***p < 0.001.

For the structural equation modelling (Fig. 2) with childhood adversity as the independent variable and executive function and processing speed as the latent, dependent variables, the model fit was very good^46,47^, CFI = 0.986, RMSEA = 0.028, and SRMR = 0.011. Higher levels of childhood adversity significantly predicted lower performance in tasks measuring executive function (β = − 0.037, *p* < 0.001), but did not predict performance in the processing speed task (β = 0.003, *p* = 0.566). Higher levels of distress and neuroticism both predicted lower executive function (β = − 0.041, *p* < 0.001 and β = − 0.028, *p* < 0.001 respectively), and higher processing speed (β = 0.020, *p* = 0.001 and β = 0.018, *p* = 0.002 respectively). From this analysis the mediated paths of adversity on cognition were tested for significance. This analysis was followed by a post hoc mediation analysis to test if the effects of childhood adversity on executive function and processing were mediated by distress and/or neuroticism. The effect of childhood adversity on executive function was partially mediated by distress (β = − 0.007, *p* < 0.001) and neuroticism (β = − 0.004, *p* < 0.001). 16.5% of the effect of childhood adversity on executive function was mediated by distress (Indirect effect/Total effect Ratio = 0.165). 10.4% of the effect of childhood adversity on executive function was mediated by neuroticism (Indirect effect/Total effect Ratio = 0.104). Significant complete mediation^48^ or indirect-only mediation^49^ was found for the effect of childhood adversity on processing speed with distress (β = 0.004, *p* = 0.001) and neuroticism (β = 0.003, *p* = 0.002) as mediators, thereby indicating that only the indirect path from childhood adversity to distress/neuroticism to processing speed was significant, whereas the direct path from childhood adversity to processing speed showed no effect. 54.3% of the effect of childhood adversity on processing speed was mediated by distress (Indirect effect/Total effect Ratio = 0.543). 48.5% of the effect of childhood adversity on processing speed was mediated by neuroticism (Indirect effect/Total effect Ratio = 0.485). Table 2 shows the path coefficients and test statistics for the direct and indirect effects in the model as well as the components.

**Table 2:** Path analysis showing the effects of childhood adversity on cognitive performance and mediation by distress and neuroticism.

Effect	β	SE	z	*p*	95% CI LB	95% CI UB
**Direct effects**						
Child adversity → Executive function	− 0.037	0.005	− 6.800	< 0.001	− 0.048	− 0.026
Child adversity → Processing speed	0.003	0.005	0.570	0.566	− 0.007	0.013
**Indirect effects**						
Child adversity → Distress → Executive function^a^	− 0.007	0.001	− 6.691	< 0.001	− 0.010	− 0.005
Child adversity → Neuroticism → Executive function^a^	− 0.004	0.001	− 4.591	< 0.001	− 0.006	− 0.002
Child adversity → Distress → Processing speed^b^	0.004	0.001	3.421	0.001	0.001	0.006
Child adversity → Neuroticism → Processing speed^b^	0.003	0.001	3.162	0.002	0.001	0.004
**Components**						
Child adversity → Distress	0.177	0.004	46.610	< 0.001	0.170	0.184
Child adversity → Neuroticism	0.152	0.004	39.920	< 0.001	0.144	0.159
Distress → Executive function	− 0.041	0.006	− 6.760	< 0.001	− 0.053	− 0.029
Neuroticism → Executive function	− 0.028	0.006	− 4.620	< 0.001	− 0.040	− 0.016
Distress → Processing speed	0.020	0.006	3.430	0.001	0.008	0.031
Neuroticism → Processing speed	0.018	0.006	3.170	0.002	0.007	0.029

Results are adjusted for covariates (sex, age, Townsend deprivation index, years of education).

*β* Standardized path coefficient, *SE* standard error, *CI* confidence interval, *LL* lower limit, *UL* upper limit.

^a^Partial mediation.

^b^Complete (in-direct only) mediation.

### Effects of adulthood adversity on cognitive performance

#### Direct effects model

**Figure 3:**
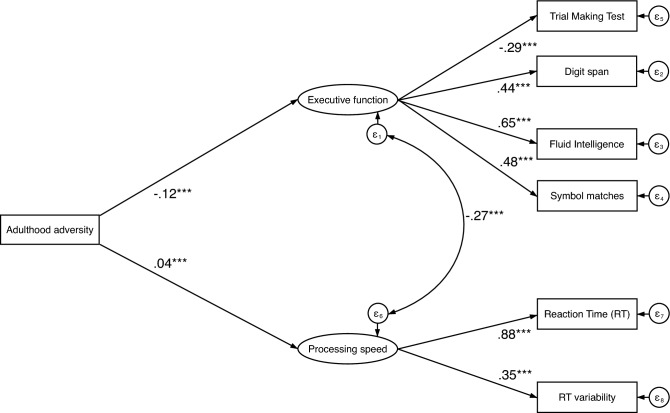
Path diagram showing the relationship between variables entered in the structural equation modelling with adulthood adversity as the independent variable, executive function and processing speed as latent dependent variables. *Note:* Path-coefficients are standardized coefficients. Covariates not shown for display purposes. Covariates include sex, age, Townsend deprivation index, and education. *p < 0.05, **p < 0.01, ***p < 0.001.

A direct effects model (Fig. 3) with adulthood adversity as the independent variable and executive function and processing speed as the latent, dependent variables had good model fit, CFI = 0.930, RMSEA = 0.050, and SRMR = 0.027. There were moderate direct effects of adulthood adversity on executive function (β = − 0.119, *p* < 0.001) and processing speed (β = 0.040, *p* < 0.001).

#### Indirect effects model

**Figure 4:**
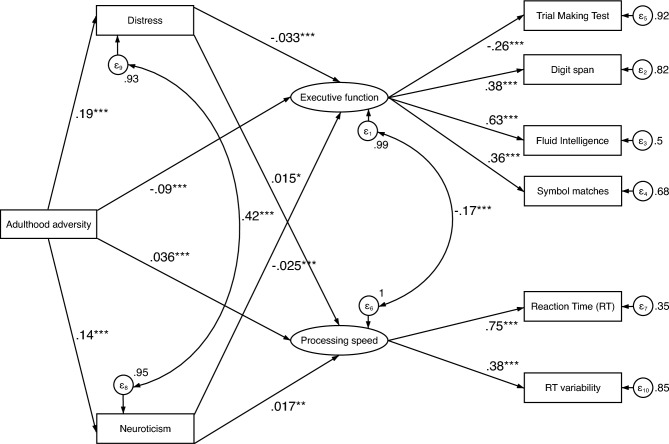
Path diagram showing the relationship between variables entered in the structural equation modelling with adulthood adversity as the independent variable, executive function and processing speed as latent dependent variables and distress and neuroticism as mediators. *Note:* Path-coefficients are standardized coefficients. Covariates not shown for display purposes. Covariates include sex, age, Townsend deprivation index, and education. *p < 0.05, **p < 0.01, ***p < 0.001.

For the SEM (Fig. 4) with adulthood adversity as the independent variable and executive function and processing speed as the latent, dependent variables, the model fit was very good^46,47^, CFI = 0.987, RMSEA = 0.028, and SRMR = 0.011. Higher levels of adulthood adversity significantly predicted lower performance in tasks measuring executive function (β = − 0.090, *p* < 0.001) and higher processing speed (β = 0.036, *p* < 0.001). Higher levels of distress and neuroticism both predicted executive function (β = − 0.033, *p* < 0.001 and β = − 0.025, *p* < 0.001 respectively) and higher processing speed (β = 0.015, *p* = 0.010 and β = 0.017, *p* = 0.003 respectively). From this analysis the significance of the mediated paths of adversity on cognition were tested for significance. The effect of adulthood adversity on executive function was partially mediated by distress (β = − 0.006, *p* < 0.001) and neuroticism (β = − 0.003, *p* < 0.001). 6.6% of the effect of adult adversity on executive function was mediated by distress (Indirect effect/Total effect Ratio = 0.066). 3.7% of the effect of adult adversity on executive function was mediated by neuroticism (Indirect effect/Total effect Ratio = 0.037). Similarly, the effect of adulthood adversity on processing speed was partially mediated by distress (β = 0.003, *p* = 0.011) and neuroticism (β = 0.002, *p* = 0.003). 7.1% of the effect of adult adversity on processing speed was mediated by distress (Indirect effect/Total effect Ratio = 0.071). 6% of the effect of adult adversity on processing speed was mediated by neuroticism (Indirect effect/Total effect = 0.060). Table 3 shows the path coefficients and test statistics for the direct and indirect effects in the model as well as the components.

**Table 3:** Path analysis showing the effects of adult adversity on cognitive performance and mediation by distress and neuroticism.

Effect	β	SE	z	*p*	95% CI LB	95% CI UB
Direct effects						
Adult adversity → Executive function	− 0.090	0.006	− 16.130	< 0.001	− 0.101	− 0.079
Adult adversity → Processing speed	0.036	0.005	6.850	< 0.001	0.026	0.047
Indirect effects						
Adult adversity → Distress → Executive function^a^	− 0.006	0.001	− 5.441	< 0.001	− 0.009	− 0.004
Adult adversity → Neuroticism → Executive function^a^	− 0.003	0.001	− 4.116	< 0.001	− 0.005	− 0.002
Adult adversity → Distress → Processing speed^a^	0.003	0.001	2.558	0.011	0.001	0.005
Adult adversity → Neuroticism → Processing speed^a^	0.002	0.001	2.970	0.003	0.001	0.004
Components						
Adult adversity → Distress	0.190	0.004	49.130	< 0.001	0.182	0.197
Adult adversity → Neuroticism	0.139	0.004	35.360	< 0.001	0.131	0.147
Distress → Executive function	− 0.033	0.006	− 5.470	< 0.001	− 0.045	− 0.021
Neuroticism → Executive function	− 0.025	0.006	− 4.140	< 0.001	− 0.037	− 0.013
Distress → Processing speed	0.015	0.006	2.560	0.010	0.003	0.026
Neuroticism → Processing speed	0.017	0.006	2.980	0.003	0.006	0.028

Results are adjusted for covariates (sex, age, Townsend deprivation index, years of education).

*β* standardized path coefficient, *SE* standard error, *CI* confidence interval, *LL* lower limit, *UL* upper limit.

^a^Partial mediation.

^b^Complete mediation.

## Discussion

Here we have investigated whether the deleterious effect of childhood adversity and adulthood adversity on cognition was mediated by neuroticism or affect-related distress in the UK Biobank, a large cohort study. Childhood adversity showed a significant direct effect on executive function; however, childhood adversity did not show a direct effect on processing speed. By contrast, adult adversity showed significant direct effects on both executive function and processing speed. We found that the negative effect of childhood adversity on executive function was partially mediated by distress and neuroticism, whereas childhood adversity did not show a direct effect on processing speed. The effect of childhood adversity on processing speed was only apparent when considering the indirect path with neuroticism and distress as significant complete mediators. For adult adversity, our findings indicate that the detrimental effects of adult adversity on executive function and processing speed were partially mediated by distress and neuroticism.

Our findings are consistent with previous research that showed that adversity negatively affects cognitive performance in a variety of cognitive tasks^6,8,9,50^. Our findings add to what is known from previous research that the detrimental effect of adversity on cognition was mediated by neuroticism and distress. These findings are important because understanding the mechanisms through which adversity affects cognitive performance opens the door to designing interventions that support cognitive health in individuals who experienced adversity in childhood or adulthood.

The findings from the exploratory factor analysis revealed two factors that are theoretically meaningful in the context of cognitive development and decline in adulthood. The factor “processing speed” can be linked to the processing-speed theory of adult age differences in cognition that suggest an association between increased age in adulthood and a decrease in the speed with which cognitive processes can be executed^51^. Processing speed has been linked to brain integrity^52^ and more specifically to white matter integrity^53^. In light of these theories, the effect of adversity on processing speed may reflect an accelerated aging process in response to the experience of adversity. Indeed, pervious research showed that child adversity was associated with accelerated aging^54^. The factor “executive function” can be linked to the prefrontal-executive theory that asserts that structural and functional changes in prefrontal cortex lead to declines in executive function, which in turn leads to lower cognitive performance^55,56^. The prefrontal cortex is sensitive to stress and particularly vulnerable during development, which is the reason why early life adversity may impair development of the prefrontal cortex^57^.

Our study has several strengths: First, the study’s sample size was large. Second, IRT was used to optimise the distress and neuroticism mediators. Compared to summated test scores, IRT has the advantage of improved precision and reliability through the identification and deletion of misfitting items^58^. Third, the analysis was adjusted for a range of covariates.

The present study has several limitations: Childhood adversity was assessed retrospectively self-report and might be subject to retrospective memory bias. Indeed, research indicated that high distress at recall was associated with a greater recall frequency of potentially traumatic events^59^. However, more recent research found that retrospectively reported child abuse was not biased by depression in adulthood^60^. Since we have found similar findings for the mediating role of distress and neuroticism for both childhood adversity and adulthood adversity on cognition, it may be unlikely that our findings are the result of a retrospective memory bias.

Another limitation resulting from the cross-sectional nature of the study design is that the mediators were acquired at approximately the same time as the independent and dependent variables in the mediation analysis model. Due to the implied causation of the mediation analysis, mediators should ideally be antecedents of the dependent variables and acquired before the dependent variables in a longitudinal design^61^. This was not possible in our analysis. However, the PHQ-9 from which our distress score was derived was previously found to be a proxy of lifetime depression^62^. Similarly, neuroticism remains rather stable in middle and older adulthood^63^. Thus, it seems to be reasonable to conclude that the distress score and neuroticism score from the UK Biobank may still serve as a proxy of past distress and neuroticism in mediation analysis. However, it should also be noted that the analysis does not shed light on the directionality of the association between distress/neuroticism and cognition and the association could be reversed.

Since we have seen here in a large and heterogenous cohort that the negative effect of adversities on cognition was mediated by neuroticism and mental-health related distress, this suggests that evidence-based interventions in clinical practice and policy-making can be specifically targeted at early mental health support. Interventions lowering mental-health-related distress or negative affectivity may have the potential to support cognitive health in individuals who experienced adversity in childhood or adulthood. Further experimental research investigating interventions for cognitive health is needed to confirm this implication.

Future research is required to investigate distress and neuroticism as mediators for the effect of adversity on cognition using a longitudinal design. Longitudinal designs have the advantage that (1) childhood adversity can be measured irrespective of any retrospective memory bias, (2) mediators in longitudinal designs are “true antecedents”, (3) pre-/post-score comparisons on the dependent variables measuring cognitive health can reduce inter-individual variability in cognition. Future research should also investigate a broader range of mediators for the effect of adversity on cognition.

In conclusion, our study demonstrated that adulthood adversity negatively impacted both performances in executive function and processing speed. Distress and neuroticism mediated the detrimental effect of childhood and adulthood adversity on cognition. Childhood adversity only negatively affected executive function, whereas lower processing speed was only found when considering the indirect path with distress and neuroticism as mediators.

## Methods

### Design

Details of the design, participants, procedure and ethics of the UK Biobank study are available elsewhere^64^. The UK Biobank is a large, population-based study that involved the recruitment of 502,665 participants and the collection of comprehensive baseline data^64^. Ethical approval was granted to UK Biobank from the Research Ethics Committee—REC reference Ref 11/NW/0382 (approval letter dated 17th June 2011)^64^. All participants gave written informed consent prior to their participation. The UK Biobank study was conducted in accordance with the Declaration of Helsinki.

### Materials

#### Adversity

Separate adversity scores were used for childhood and adulthood adversity as previous research indicated differential effects of adversity on cognition across the lifespan^7^. The composite score for childhood adversity was derived from items from the Childhood Trauma Screener (CTS-5)^65^ that were scored on a 5-point Likert scale: *Never true (0), Rarely true (1), Sometimes true (2), Often (3), Very often true (4)*. The following items were used to yield the composite childhood adversity score: “When I was growing up… I felt that someone in my family hated me”, “When I was growing up… People in my family hit me so hard that it left me with bruises or marks”, “When I was growing up… Someone molested me (sexually)”, and “When I was growing up… I felt loved”. The composite score of childhood adversity was computed by summing up these four items and ranges from 0 to 16, with higher scores indicating higher levels of adversity.

The adulthood adversity score was derived from items that were adapted from the British Crime Survey^66^ that were also scored on a 5-point Likert scale. The following items were used to yield the composite adulthood adversity score: “Since I was sixteen… A partner or ex-partner sexually interfered with me, or forced me to have sex against my wishes”, “Since I was sixteen… A partner or ex-partner deliberately hit me or used violence in any other way”, “Since I was sixteen… A partner or ex-partner repeatedly belittled me to the extent that I felt worthless”, and “Since I was sixteen… I have been in a confiding relationship”. The composite score of adulthood adversity was computed by summing up these four items. This composite score of adulthood adversity ranges from 0 to 16, with higher scores indicating higher levels of adversity.

#### Distress

The PHQ-ADS scale^21^ was used to measure affect-related distress and an optimised latent trait variable was derived using item-response theory. The procedure used to derive the latent trait variable involved discarding misfitting items from the scale and applying an item-response theory model on 7 items with good model fit as previously described in more detail^67^. Data from three trials previously showed that the PHQ-ADS has high internal reliability and high construct and convergent validity^21^.

#### Neuroticism

The Eysenck Personality Questionnaire^68^ was used to measure neuroticism and an optimised ed latent trait variable was derived following a procedure previously described^69^.

#### Cognition

Details of the cognitive tasks selected for this study are reported in the supplementary materials. In brief, we used the digit span task assessing numeric short-term memory, the fluid intelligence test, symbol matches task measuring complex attention, and trail-making tasks that measure executive function, visual scanning, and working memory as well as the average and variability of reaction times in the snap game task.

#### Covariates

Models were adjusted for age, gender, Townsend deprivation index (TDI) and education (see supplementary materials, methods section for details).

### Analytic strategy

UK Biobank data for this analysis (application 15697) were uploaded onto the Dementias Platform UK (DPUK) Data Portal^70^ and analysed using STATA SE 17.0^71^. An SEM was fitted to the data with childhood adversity or adulthood adversity as the independent variable, cognitive performance as the dependent variable and distress and neuroticism as mediators. The Stata package *medsem*^72^ was used to test for mediational hypotheses^48,49^ using Sobel estimators^73^. The goodness of fit of the SEM was assessed using the Comparative Fit Index (CFI), Root Mean Squared Error of Approximation (RMSEA), and Standardized Root Mean Square Residual (SRMR).

### Supplementary Information

Supplementary Information.

## Data Availability

The dataset(s) supporting the conclusions of this article is(are) available in the Dementias Platform UK (DPUK) Data Portal repository, https://portal.dementiasplatform.uk/. Access to the data can be requested through UK Biobank (https://www.ukbiobank.ac.uk/enable-your-research/apply-for-access).

## References

[1] Patterson ML, Moniruzzaman A, Somers JM (2014). Setting the stage for chronic health problems: Cumulative childhood adversity among homeless adults with mental illness in Vancouver, British Columbia. BMC Public Health.

[2] Racine N, Eirich R, Dimitropoulos G, Hartwick C, Madigan S (2020). Development of trauma symptoms following adversity in childhood: The moderating role of protective factors. Child Abuse Negl..

[3] Reid JA, Baglivio MT, Piquero AR, Greenwald MA, Epps N (2017). Human trafficking of minors and childhood adversity in Florida. Am. J. Public Health.

[4] U.S. Department of Health & Human Services. *Child Maltreatment*. https://www.acf.hhs.gov/sites/default/files/documents/cb/cm2021.pdf (2021).

[5] Nelson CA (2020). Adversity in childhood is linked to mental and physical health throughout life. BMJ.

[6] Aartsen MJ (2019). Advantaged socioeconomic conditions in childhood are associated with higher cognitive functioning but stronger cognitive decline in older age. Proc. Natl. Acad. Sci. U. S. A..

[7] Kuenzi, M. *The Impact of Life Course Adversity on Later Life Cognition*. 10.13097/archive-ouverte/unige:164543 (2022).

[8] Richards M, Wadsworth MEJ (2004). Long term effects of early adversity on cognitive function. Arch. Dis. Child..

[9] Shonkoff JP, Garner AS (2012). The lifelong effects of early childhood adversity and toxic stress. Pediatrics.

[10] Hostinar CE, Stellern SA, Schaefer C, Carlson SM, Gunnar MR (2012). Associations between early life adversity and executive function in children adopted internationally from orphanages. Proc. Natl. Acad. Sci. U. S. A..

[11] Cristofori, I., Cohen-Zimerman, S. & Grafman, J. Chapter 11—Executive functions. In *The Frontal Lobes* (eds. D’Esposito, M. & Grafman, J. H. B. T.-H. of C. N.) vol. 163 197–219 (Elsevier, 2019). 10.1016/B978-0-12-804281-6.00011-2 31590731

[12] Künzi M (2022). Cumulative life course adversity, mental health, and cognition in the UK biobank. Sci. Rep..

[13] Costa PT, McCrae RR (1985). The NEO Personality Inventory: Manual form S and form R.

[14] Boillat C (2017). Neuroticism as a risk factor for child abuse in victims of childhood sexual abuse. Child Abuse Negl..

[15] Da Silva Coelho C (2022). Higher levels of neuroticism in older adults predict lower executive functioning across time: the mediating role of perceived stress. Eur. J. Ageing.

[16] Saylik R, Szameitat AJ, Cheeta S (2018). Neuroticism related differences in working memory tasks. PLoS One.

[17] Sutin AR, Stephan Y, Luchetti M, Terracciano A (2019). Five-factor model personality traits and cognitive function in five domains in older adulthood. BMC Geriatr..

[18] Eysenck, H. J. The biological basis of personality. In *Personality Structure and Measurement (Psychology Revivals)* (eds Eysenck, H. J. & Eysenck, S. B. G.), 49–62 (Routledge, 2013).

[19] Murphy F (2022). Childhood trauma, the HPA axis and psychiatric illnesses: A targeted literature synthesis. Front. Psychiatry.

[20] Beishon LC (2022). The role of the autonomic nervous system in cerebral blood flow regulation in dementia: A review. Auton. Neurosci..

[21] Kroenke K (2016). The patient health questionnaire anxiety and depression scale (PHQ-ADS): Initial validation in three clinical trials. Psychosom. Med..

[22] Viertiö S (2021). Factors contributing to psychological distress in the working population with a special reference to gender difference. BMC Public Health.

[23] Groen RN (2020). Comorbidity between depression and anxiety: Assessing the role of bridge mental states in dynamic psychological networks. BMC Med..

[24] Hanel G (2009). Depression, anxiety, and somatoform disorders: Vague or distinct categories in primary care? Results from a large cross-sectional study. J. Psychosom. Res..

[25] ter Meulen WG (2021). Depressive and anxiety disorders in concert: A synthesis of findings on comorbidity in the NESDA study. J. Affect. Disord..

[26] American Psychiatric Association (2013). Diagnostic and Statistical Manual of Mental Disorders (DSM-5®).

[27] Levitan RD, Rector NA, Sheldon T, Goering P (2003). Childhood adversities associated with major depression and/or anxiety disorders in a community sample of Ontario: Issues of co-morbidity and specificity. Depress. Anxiety.

[28] McLaughlin TP, Khandker RK, Kruzikas DT, Tummala R (2006). Overlap of anxiety and depression in a managed care population: Prevalence and association with resource utilization. J. Clin. Psychiatry.

[29] Rodriguez BF (2004). Frequency and patterns of psychiatric comorbidity in a sample of primary care patients with anxiety disorders. Compr. Psychiatry.

[30] Goodwin GM (2015). The overlap between anxiety, depression, and obsessive-compulsive disorder. Dialogues Clin. Neurosci..

[31] Marackova M (2016). The impact of childhood adversities on anxiety and depressive disorders in adulthood. Neuro Endocrinol. Lett..

[32] Hovens JGFM (2010). Childhood life events and childhood trauma in adult patients with depressive, anxiety and comorbid disorders vs. controls. Acta Psychiatr. Scand..

[33] Gilbert R (2009). Burden and consequences of child maltreatment in high-income countries. Lancet.

[34] Kessler RC (2010). Childhood adversities and adult psychopathology in the WHO World Mental Health Surveys. Br. J. Psychiatry.

[35] Chamberlain SR, Sahakian BJ (2006). The neuropsychology of mood disorders. Curr. Psychiatry Rep..

[36] Iglesias C, Alonso M (2009). Residual symptoms in depression. Actas Esp. Psiquiatr..

[37] Wei J (2019). Late-life depression and cognitive function among older adults in the U.S.: The national health and nutrition examination survey, 2011–2014. J. Psychiatr. Res..

[38] Shimada H (2014). Depressive symptoms and cognitive performance in older adults. J. Psychiatr. Res..

[39] Rana T, Behl T, Sehgal A, Srivastava P, Bungau S (2021). Unfolding the role of BDNF as a biomarker for treatment of depression. J. Mol. Neurosci..

[40] Ferrer A (2019). BDNF genetic variants and methylation: Effects on cognition in major depressive disorder. Transl. Psychiatry.

[41] Duman RS, Monteggia LM (2006). A neurotrophic model for stress-related mood disorders. Biol. Psychiatry.

[42] Lu B, Nagappan G, Lu Y (2014). BDNF and synaptic plasticity, cognitive function, and dysfunction. Handb. Exp. Pharmacol..

[43] Sumner JA (2017). Posttraumatic stress disorder symptoms and cognitive function in a large cohort of middle-aged women. Depress. Anxiety.

[44] Schuitevoerder S (2013). A meta-analysis of cognitive functioning in older adults with PTSD. J. Anxiety Disord..

[45] Hamilton LC (2003). Statistics with Stata: Updated for Version 7.

[46] Hu L, Bentler PM (1999). Cutoff criteria for fit indexes in covariance structure analysis: Conventional criteria versus new alternatives. Struct. Equ. Model. A Multidiscip. J..

[47] Kline RB (2016). Principles and Practice of Structural Equation Modeling.

[48] Baron RM, Kenny DA (1986). The moderator-mediator variable distinction in social psychological research: Conceptual, strategic, and statistical considerations. J. Pers. Soc. Psychol..

[49] Zhao X, Lynch JG, Chen Q (2010). Reconsidering Baron and Kenny: Myths and truths about mediation analysis. J. Consum. Res..

[50] Künzi M (2021). The relationship between life course socioeconomic conditions and objective and subjective memory in older age. Brain Sci..

[51] Salthouse TA (1996). The processing-speed theory of adult age differences in cognition. Psychol. Rev..

[52] Birren JE, Fisher LM (1995). Aging and speed of behavior: Possible consequences for psychological functioning. Annu. Rev. Psychol..

[53] Madden DJ (2004). Diffusion tensor imaging of adult age differences in cerebral white matter: Relation to response time. Neuroimage.

[54] Bourassa KJ (2023). Which types of stress are associated with accelerated biological aging? Comparing perceived stress, stressful life events, childhood adversity, and posttraumatic stress disorder. Psychosom. Med..

[55] Albinet CT, Boucard G, Bouquet CA, Audiffren M (2012). Processing speed and executive functions in cognitive aging: How to disentangle their mutual relationship?. Brain Cogn..

[56] West RL (1996). An application of prefrontal cortex function theory to cognitive aging. Psychol. Bull..

[57] McEwen BS, Morrison JH (2013). The brain on stress: vulnerability and plasticity of the prefrontal cortex over the life course. Neuron.

[58] Henning G (1984). Advantages of latent trait measurement in language testing. Lang. Test..

[59] Lalande KM, Bonanno GA (2011). Retrospective memory bias for the frequency of potentially traumatic events: A prospective study. Psychol. Trauma Theory Res. Pract. Policy.

[60] Pinto Pereira SM, Rogers NT, Power C (2021). Adult retrospective report of child abuse and prospective indicators of childhood harm: A population birth cohort study. BMC Med..

[61] Jose PE (2013). Doing Statistical Mediation and Moderation.

[62] Cannon DS (2007). The PHQ-9 as a brief assessment of lifetime major depression. Psychol. Assess..

[63] Steunenberg B, Twisk J, Beekman A, Deeg D, Kerkhof A (2005). Stability and change of neuroticism in aging. J. Gerontol. B. Psychol. Sci. Soc. Sci..

[64] Sudlow C (2015). UK biobank: An open access resource for identifying the causes of a wide range of complex diseases of middle and old age. PLoS Med..

[65] Glaesmer H (2013). The childhood trauma screener (CTS): Development and validation of cut-off-scores for classificatory diagnostics. Psychiatr. Prax..

[66] Khalifeh H, Oram S, Trevillion K, Johnson S, Howard LM (2015). Recent intimate partner violence among people with chronic mental illness: Findings from a national cross-sectional survey. Br. J. Psychiatry.

[67] Pflanz CP, Gallacher J, Bauermeister S (2024). A psychometric evaluation of the 16-item PHQ-ADS concomitant anxiety and depression scale in the UK biobank using item response theory. J. Affect. Disord..

[68] Eysenck SB, Eysenck HJ, Barrett P (1985). A revised version of the psychoticism scale. Pers. Individ. Differ..

[69] Bauermeister S, Pflanz CP, Gallacher J (2022). Adapting the Eysenck personality questionnaire-revised neuroticism scale for use in epidemiologic studies: A psychometric evaluation using item response theory in the UK Biobank. bioRxiv.

[70] Bauermeister S (2019). The dementias platform UK (DPUK) data portal. Eur. J. Epidemiol..

[71] StataCorp. Stata Statistical Software College Station. at www.stata.com (2021).

[72] Mehmetoglu M (2018). medsem: A Stata package for statistical mediation analysis. Int. J. Comput. Econ. Econom..

[73] Sobel ME (1987). Direct and indirect effects in linear structural equation models. Sociol. Methods Res..

