# Supplementary material for: Distress and neuroticism as mediators of the effect of childhood and adulthood adversity on cognitive performance in the UK Biobank study
Source: Sci Rep. 2024 Apr 6;14:8108. doi: 10.1038/s41598-024-58510-z (PMC10998912; doi:10.1038/s41598-024-58510-z)
Supplement: Supplementary file 1 — Supplementary Information. [file 41598_2024_58510_MOESM1_ESM.docx]

**Supplementary material**

**Supplementary methods**

**Cognition**

For the purpose of the present study, we selected the fluid intelligence test, trail making task, symbol digit substitution task, and the numeric memory test from the cognitive function online follow-up as our tasks of interest because they were acquired at the same time-point (online follow-up) as the PHQ-ADS scale. The pairs matching task from the online follow-up was not used in this specific subsample due to floor effects. We also used the 'Snap' card-game task as a reaction time task that was acquired during the baseline assessment centre. The prospective memory task from the baseline assessment was not used for the present analysis because it is a binary outcome measure and therefore not suitable for linear structural equation modelling. The matrix pattern completion task, tower rearranging task, picture vocabulary test, and paired associate learning task were not used to assess cognitive function because these tasks were not introduced in the UK Biobank until 2016 at the imaging clinics, which is a considerable period of time later than the online follow-up questionnaire. The lights pattern memory task and the word production test were not used because they were only performed in the pilot phase of the study and are only available for a subset of participants.

From the Trial Making Test that measures executive functioning (Reitan, 1958), a ratio score was calculated by dividing the TMT Part B (TMTB) by the TMT Part A (TMTA) (Arbuthnott & Frank, 2000), with the TMTA measuring the time in seconds to correctly connect the numbers 1 to 25 in ascending order, and the TMTB measuring the time in seconds to correctly connect numbers and letters in ascending and alphabetic alternating between number and letter. This ratio serves as an indicator of the cognitive flexibility component of executive function (Arbuthnott & Frank, 2000). During the working memory task, the participant was shown a 2-digit number to remember, the number then disappeared and after a short while, they were asked to enter the number onto the screen. The number became one digit longer each time they remembered correctly up to a maximum of 12 digits. The longest number correctly recalled serves as an indicator of numeric working memory. The fluid intelligence test measures the capacity to solve problems that require logic and reasoning ability, independent of acquired knowledge. It was a bespoke test only used for UK Biobank. During the symbol digit substitution test participants were presented with a series of symbols that they were asked to match to numbers according to a key. The number of correct symbol digit matches was used as the task score. The number of correct symbol digit matches is the number of times a participant correctly matched a symbol with a digit summed across all the rounds of the test, excluding the initial 8 items that correspond to the training round.

The mean reaction time and reaction time variability were derived from the snap card game. Rounds 5 to 11 were used thereby excluding the first four rounds that were used as training round and reaction times under 50ms were discarded because they were due to anticipation rather than reaction, and reaction times over 2000ms were discarded because the cards had disappeared by then. Participants with more than 3 missing rounds in the snap task were also excluded from the analysis. The intra-individual variability in reaction time was calculated following a procedure described by (Haynes et al., 2017) by dividing the intra-individual standard deviation in reaction time by the intra-individual mean reaction time.

Cognitive performance indicators were modelled using a two-step procedure: First, an exploratory factor analysis using an orthogonal varimax rotation was conducted to investigate the dimensionality of the cognitive data in the UK Biobank and to determine which cognitive scores measure the same latent construct. Second, using the factor dimensionality information from the exploratory factor analysis, cognitive performance was modelled as two latent variables that capture distinct aspects of cognitive functioning (Figures 1 and 2).

Only participants without any missing values in the variables and items used to create the variables of interest were included in the model. Such a complete-case analysis was suitable because the data were missing at random (Karahalios et al., 2012). Analyses were run listwise. Since the UK Biobank sample was large enough without missing values, power was not an issue, and the values were missing at random, listwise deletion was deemed to be a reasonable strategy (Kang, 2013). The structural equation model on childhood adversity included 64,051 participants. The structural equation model on adulthood adversity included 63,360 participants.

**Covariates**

Age, gender (coded 0 for women and 1 for men), the Townsend deprivation index (Townsend et al., 1988) and the age at which subjects completed full-time education were included as covariates in the structural equation model. Missing data in the age when full-time education was completed were imputed using the Qualifications variable from the UK Biobank, using the following average ages corresponding to the qualifications: 21 years for College/University degree or other professional qualifications (e.g. nursing, teaching), 17 years for A-levels/AS-levels or NVQ/HND/HNC or equivalent, and 15 years for O-levels/GCSE and CSE or equivalent.

**Secondary analyses**

A secondary analysis was conducted to further elucidate the findings from the SEMs presented in the results section. A series of single mediation single outcome SEMs were conducted with child or adult adversity as predictor, distress or neuroticism as mediators, and processing speed or executive function as outcome variables. The rationale behind carrying out this simplified mediation analysis was to investigate if there were suppression or enhancement effects in the dual mediation dual outcome SEMs, wherein one mediator or outcome variable entered in the model can either suppress or enhance the significance of the other mediator or outcome variable.

A further secondary analysis was conducted to investigate which indicator variables were driving the mediated effects of adversity on the latent outcome variables, executive function and processing speed. A series for SEMs was conducted with child adversity or adult adversity as predictor, distress or neuroticism as mediators, and digit span performance, fluid intelligence, correct symbol matches, performance on the trial making task, average reaction time, and reaction time variability as observed outcome variables.

**Supplementary results**

**Spearman correlations between all variables entered in the structural equation model**

Supplementary Table S2 shows the Spearman correlations with a Bonferroni correction between all variables entered in the structural equation model including covariates. Higher levels of childhood adversity were significantly correlated with higher levels of distress, higher levels of neuroticism, higher Townsend index, fewer years of education, being younger, and lower performance in tasks requiring executive function. Higher levels of adulthood adversity were significantly correlated with higher levels of distress, higher levels of neuroticism, higher Townsend index, fewer years of education, being younger, being female, lower performance in tasks requiring executive function, and longer reaction times in processing speed tasks.

**Effects of covariates in the childhood adversity model**

For the covariates, older individuals showed lower performance in cognitive tasks, lower levels of distress and neuroticism, and lower levels of childhood adversity. Being male was associated with higher performance in cognitive tasks, lower levels of distress and neuroticism, and lower levels of childhood adversity. Greater degrees of deprivation were associated with worse performance in cognitive tasks, higher levels of distress and neuroticism, and higher levels of childhood adversity. Finally, more years of full-time education were associated with better performance in cognitive tasks, lower levels of distress and neuroticism, and lower levels of childhood adversity. The path coefficients and test statistics for covariates are displayed in Supplementary Table S3.

**Effects of covariates in the adulthood adversity model**

For the covariates, older individuals showed lower performance in cognitive tasks, lower levels of distress and neuroticism, and lower levels of adulthood adversity. Being male was associated with higher performance in the digit span task, the fluid intelligence task and the snap task, as well as lower levels of distress and neuroticism and lower levels of adulthood adversity. Greater degrees of deprivation were associated with worse performance in cognitive tasks, higher levels of neuroticism, and higher levels of adulthood adversity. Finally, more years of full-time education were associated with better performance in cognitive tasks, lower levels of distress and neuroticism, and lower levels of adulthood adversity. The path coefficients and test statistics for covariates are displayed in Supplementary Table S4.**Secondary analysis: Single mediator single outcome SEMs**

Overall, the single mediator single outcome SEMs showed evidence for the role of distress and neuroticism as mediators of the effect of childhood adversity on executive function and evidence for the role of distress and neuroticism as mediators of the effect of adulthood adversity on both executive function and processing speed (Tables S5, S6). These findings were similar to the findings from the dual mediation dual outcome SEMs presented in the results section. Notably, the structural equation models with child adversity as predictor, distress or neuroticism as mediators, and processing speed as outcome variable showed a significant direct effect of child adversity on processing speed that was not found in the corresponding dual mediation dual outcome SEM (Table S5). This finding indicates that there is a direct effect of child adversity on processing speed, however, this effect becomes negligible when the effect of child adversity on executive function is taken into account in the model specification. Due to the presence of this direct effect, the single mediator single outcome SEM found evidence for partial mediation of the effect of childhood adversity on processing speed when compared with complete evidence in the dual mediator dual outcome SEM.

**Secondary analysis: SEMs with observed outcome variables**

The series of SEMs with distress and neuroticism as mediators and observed outcome variables revealed evidence of mediation for the effects of child adversity or adult adveristy on numeric digit span, symbol matches, trail making test performance, and reaction time (Supplementary Tables 7.8). No evidence of mediation by neuroticism was found for the outcome variables fluid intelligence and reaction time variability. By contrast, distress seemed to mediate the effects of childhood adversity or adulthood adversity on fluid intelligence and reaction time variability, but the effects were small.

**Supplementary Tables**

**Supplementary Table S1**

*Frequency and percent of childhood adversity and adulthood adversity for each point of the adversity composite score*

| Adversity composite score | Child adversity | | Adult adversity | |
| --- | --- | --- | --- | --- |
|  | Frequency | Percent | Frequency | Percent |
| 0 | 27,644 | 43.16 | 25,807 | 40.73 |
| 1 | 14,806 | 23.12 | 10,604 | 16.74 |
| 2 | 8,818 | 13.77 | 10,202 | 16.1 |
| 3 | 4,483 | 7 | 4,918 | 7.76 |
| 4 | 3,112 | 4.86 | 6,711 | 10.59 |
| 5 | 1,753 | 2.74 | 1,687 | 2.66 |
| 6 | 1,230 | 1.92 | 1,369 | 2.16 |
| 7 | 792 | 1.24 | 666 | 1.05 |
| 8 | 551 | 0.86 | 528 | 0.83 |
| 9 | 315 | 0.49 | 290 | 0.46 |
| 10 | 217 | 0.34 | 200 | 0.32 |
| 11 | 145 | 0.23 | 112 | 0.18 |
| 12 | 80 | 0.12 | 141 | 0.22 |
| 13 | 46 | 0.07 | 62 | 0.1 |
| 14 | 34 | 0.05 | 39 | 0.06 |
| 15 | 10 | 0.02 | 14 | 0.02 |
| 16 | 15 | 0.02 | 10 | 0.02 |
| Total | 64,051 | 100 | 63,360 | 100 |

**Supplementary Table S2.**

*Spearman correlations with a Bonferroni correction for all the variables in the structural equation models on childhood adversity and adult adversity including covariates.*

|  | Child  adversity | Adult  adversity | Gender | Age | TDI | Education | Neuro- ticism | Distress | Executive  function | Processing speed | Digit  span | Fluid intelli-gence | Symbol  matches | TMT | Snap  RT | RT  variability |
| --- | --- | --- | --- | --- | --- | --- | --- | --- | --- | --- | --- | --- | --- | --- | --- | --- |
| Child adversity | ⸺ |  |  |  |  |  |  |  |  |  |  |  |  |  |  |  |
| Adult adversity | 0.2774* | ⸺ |  |  |  |  |  |  |  |  |  |  |  |  |  |  |
| Gender | -0.0048 | -0.1152* | ⸺ |  |  |  |  |  |  |  |  |  |  |  |  |  |
| Age | -0.0518* | -0.0577* | 0.1041* | ⸺ |  |  |  |  |  |  |  |  |  |  |  |  |
| TDI | 0.0770* | 0.1316* | -0.0371* | -0.0970* | ⸺ |  |  |  |  |  |  |  |  |  |  |  |
| Education | -0.0510* | -0.0684* | 0.0215* | -0.1145* | 0.0013 | ⸺ |  |  |  |  |  |  |  |  |  |  |
| Neuroticism | 0.1569* | 0.1574* | -0.1177* | -0.1457* | 0.0432* | -0.0531* | ⸺ |  |  |  |  |  |  |  |  |  |
| Distress | 0.1682* | 0.1932* | -0.1284* | -0.1541* | 0.0582* | 0.0013 | 0.4373* | ⸺ |  |  |  |  |  |  |  |  |
| Executive function | -0.0516* | -0.0805* | 0.0107 | 0.0114 | -0.0007 | 0.0208* | -0.0653* | -0.0706* | ⸺ |  |  |  |  |  |  |  |
| Processing speed | 0.0119 | 0.0390* | -0.002 | -0.0325* | -0.0019 | 0.0049 | 0.0389* | 0.0415* | -0.2463* | ⸺ |  |  |  |  |  |  |
| Digit span | -0.0095 | -0.0340* | 0.0641* | -0.1057* | 0.0025 | 0.1384* | -0.0308* | -0.0145 | 0.5005* | -0.0585* | ⸺ |  |  |  |  |  |
| Fluid intelligence | -0.0322* | -0.0782* | 0.0468* | -0.1020* | -0.0159 | 0.3141* | -0.0231* | -0.01 | 0.8359* | -0.1228* | 0.2977* | ⸺ |  |  |  |  |
| Symbol matches | -0.0136 | -0.0354* | -0.0461* | -0.4432* | -0.0099 | 0.1548* | 0.0167 | 0.0247* | 0.4485* | -0.1427* | 0.1865* | 0.2981* | ⸺ |  |  |  |
| TMT | 0.0206* | 0.0262* | 0.0360* | 0.1022* | 0.013 | -0.0954* | 0.0112 | 0.0154 | -0.3305* | 0.0562* | -0.1435* | -0.2066* | -0.0981* | ⸺ |  |  |
| Snap RT | -0.0031 | 0.0341* | -0.0895* | 0.2958* | -0.0014 | -0.0792* | -0.0061 | -0.0097 | -0.1598* | 0.8962* | -0.0680* | -0.1109* | -0.2599* | 0.0634* | ⸺ |  |
| RT variability | -0.0061 | 0.0052 | -0.0219* | 0.0737* | -0.0082 | -0.0327* | -0.0007 | -0.0033 | -0.0841* | 0.4314* | -0.0335* | -0.0558* | -0.0725* | 0.0316* | 0.2606* | ⸺ |

*Note.* TDI: Townsend Deprivation Index, TMT: Trail Making Test, RT: Reaction Time. * p ≤ .001.

**Supplementary Table S3**

*Standardized path coefficients and test statistics for covariates in the childhood adversity structural equation model*

| Dependent variable | Covariate | β | SE | z | p | 95% CI | |
| --- | --- | --- | --- | --- | --- | --- | --- |
|  |  |  |  |  |  | LL | UL |
| Child adversity | TDI | 0.098 | 0.004 | 25.130 | 0.000 | 0.091 | 0.106 |
|  | Education | -0.061 | 0.004 | -15.510 | 0.000 | -0.069 | -0.053 |
|  | Age | -0.070 | 0.004 | -17.770 | 0.000 | -0.078 | -0.063 |
|  | Gender | -0.034 | 0.004 | -8.740 | 0.000 | -0.042 | -0.027 |
| Neuroticism | TDI | 0.016 | 0.004 | 4.140 | 0.000 | 0.008 | 0.024 |
|  | Education | -0.056 | 0.004 | -14.610 | 0.000 | -0.064 | -0.049 |
|  | Age | -0.124 | 0.004 | -31.950 | 0.000 | -0.131 | -0.116 |
|  | Gender | -0.095 | 0.004 | -24.700 | 0.000 | -0.102 | -0.087 |
| Distress | TDI | 0.029 | 0.004 | 7.550 | 0.000 | 0.022 | 0.037 |
|  | Education | -0.009 | 0.004 | -2.320 | 0.020 | -0.016 | -0.001 |
|  | Age | -0.129 | 0.004 | -33.710 | 0.000 | -0.137 | -0.122 |
|  | Gender | -0.104 | 0.004 | -27.440 | 0.000 | -0.112 | -0.097 |
| Digit span | TDI | -0.008 | 0.004 | -1.970 | 0.049 | -0.015 | 0.000 |
|  | Education | 0.118 | 0.004 | 30.450 | 0.000 | 0.110 | 0.126 |
|  | Age | -0.108 | 0.004 | -27.370 | 0.000 | -0.116 | -0.100 |
|  | Gender | 0.066 | 0.004 | 16.990 | 0.000 | 0.059 | 0.074 |
| Fluid intelligence | TDI | -0.026 | 0.004 | -6.900 | 0.000 | -0.033 | -0.019 |
|  | Education | 0.294 | 0.004 | 82.980 | 0.000 | 0.287 | 0.301 |
|  | Age | -0.083 | 0.004 | -21.750 | 0.000 | -0.091 | -0.076 |
|  | Gender | 0.047 | 0.004 | 12.450 | 0.000 | 0.040 | 0.054 |
| Symbol matches | TDI | -0.056 | 0.004 | -15.750 | 0.000 | -0.063 | -0.049 |
|  | Education | 0.106 | 0.004 | 29.940 | 0.000 | 0.099 | 0.113 |
|  | Age | -0.423 | 0.003 | -134.110 | 0.000 | -0.429 | -0.417 |
|  | Gender | -0.003 | 0.004 | -0.760 | 0.446 | -0.010 | 0.004 |
| TMT | TDI | 0.028 | 0.004 | 7.050 | 0.000 | 0.020 | 0.035 |
|  | Education | -0.083 | 0.004 | -21.210 | 0.000 | -0.091 | -0.076 |
|  | Age | 0.092 | 0.004 | 23.270 | 0.000 | 0.085 | 0.100 |
|  | Gender | 0.031 | 0.004 | 7.850 | 0.000 | 0.023 | 0.039 |
| Snap RT | TDI | 0.030 | 0.004 | 7.830 | 0.000 | 0.022 | 0.037 |
|  | Education | -0.042 | 0.004 | -11.090 | 0.000 | -0.050 | -0.035 |
|  | Age | 0.287 | 0.004 | 78.490 | 0.000 | 0.280 | 0.294 |
|  | Gender | -0.103 | 0.004 | -27.200 | 0.000 | -0.111 | -0.096 |
| RT variability | TDI | -0.003 | 0.004 | -0.830 | 0.408 | -0.011 | 0.004 |
|  | Education | -0.019 | 0.004 | -4.760 | 0.000 | -0.027 | -0.011 |
|  | Age | 0.077 | 0.004 | 19.290 | 0.000 | 0.069 | 0.085 |
|  | Gender | -0.028 | 0.004 | -6.960 | 0.000 | -0.035 | -0.020 |

*Note.* β: Standardized path coefficient, SE: standard error, CI: confidence Interval, LL: lower limit, UL: upper limit, TDI: Townsend Deprivation Index, TMT: Trail Making Test, RT: Reaction Time.

**Supplementary Table S4**

*Standardized path coefficients and test-statistics for covariates in the adulthood adversity structural equation model*

| Dependent variable | Covariate | β | SE | z | p | 95% CI | |
| --- | --- | --- | --- | --- | --- | --- | --- |
|  |  |  |  |  |  | LL | UL |
| Adult adversity | TDI | 0.133 | 0.004 | 34.530 | 0.000 | 0.125 | 0.140 |
|  | Education | -0.083 | 0.004 | -21.360 | 0.000 | -0.090 | -0.075 |
|  | Age | -0.050 | 0.004 | -12.680 | 0.000 | -0.058 | -0.042 |
|  | Gender | -0.143 | 0.004 | -37.250 | 0.000 | -0.150 | -0.135 |
| Neuroticism | TDI | 0.014 | 0.004 | 3.470 | 0.001 | 0.006 | 0.021 |
|  | Education | -0.055 | 0.004 | -14.070 | 0.000 | -0.062 | -0.047 |
|  | Age | -0.128 | 0.004 | -32.890 | 0.000 | -0.136 | -0.120 |
|  | Gender | -0.081 | 0.004 | -20.810 | 0.000 | -0.089 | -0.074 |
| Distress | TDI | 0.022 | 0.004 | 5.650 | 0.000 | 0.014 | 0.030 |
|  | Education | -0.004 | 0.004 | -0.960 | 0.339 | -0.011 | 0.004 |
|  | Age | -0.133 | 0.004 | -34.540 | 0.000 | -0.140 | -0.125 |
|  | Gender | -0.085 | 0.004 | -21.860 | 0.000 | -0.092 | -0.077 |
| Digit span | TDI | -0.004 | 0.004 | -1.030 | 0.305 | -0.012 | 0.004 |
|  | Education | 0.118 | 0.004 | 30.240 | 0.000 | 0.110 | 0.126 |
|  | Age | -0.108 | 0.004 | -27.340 | 0.000 | -0.116 | -0.100 |
|  | Gender | 0.063 | 0.004 | 16.050 | 0.000 | 0.055 | 0.071 |
| Fluid intelligence | TDI | -0.021 | 0.004 | -5.570 | 0.000 | -0.029 | -0.014 |
|  | Education | 0.290 | 0.004 | 81.440 | 0.000 | 0.283 | 0.297 |
|  | Age | -0.083 | 0.004 | -21.650 | 0.000 | -0.091 | -0.076 |
|  | Gender | 0.041 | 0.004 | 10.620 | 0.000 | 0.033 | 0.048 |
| Symbol matches | TDI | -0.054 | 0.004 | -15.100 | 0.000 | -0.061 | -0.047 |
|  | Education | 0.105 | 0.004 | 29.350 | 0.000 | 0.098 | 0.112 |
|  | Age | -0.422 | 0.003 | -132.750 | 0.000 | -0.428 | -0.415 |
|  | Gender | -0.006 | 0.004 | -1.730 | 0.084 | -0.013 | 0.001 |
| TMT | TDI | 0.025 | 0.004 | 6.440 | 0.000 | 0.018 | 0.033 |
|  | Education | -0.081 | 0.004 | -20.550 | 0.000 | -0.089 | -0.073 |
|  | Age | 0.092 | 0.004 | 23.160 | 0.000 | 0.084 | 0.100 |
|  | Gender | 0.033 | 0.004 | 8.350 | 0.000 | 0.025 | 0.041 |
| Snap RT | TDI | 0.027 | 0.004 | 7.050 | 0.000 | 0.020 | 0.035 |
|  | Education | -0.039 | 0.004 | -10.180 | 0.000 | -0.046 | -0.031 |
|  | Age | 0.287 | 0.004 | 78.140 | 0.000 | 0.280 | 0.295 |
|  | Gender | -0.099 | 0.004 | -25.810 | 0.000 | -0.107 | -0.092 |
| RT variability | TDI | -0.004 | 0.004 | -0.950 | 0.340 | -0.012 | 0.004 |
|  | Education | -0.017 | 0.004 | -4.350 | 0.000 | -0.025 | -0.010 |
|  | Age | 0.077 | 0.004 | 19.040 | 0.000 | 0.069 | 0.085 |
|  | Gender | -0.026 | 0.004 | -6.440 | 0.000 | -0.034 | -0.018 |

*Note.* β: Standardized path coefficient, SE: standard error, CI: confidence Interval, LL: lower limit, UL: upper limit, TDI: Townsend Deprivation Index, TMT: Trail Making Test, RT: Reaction Time.

**Table S5.**

*Path analyses using simplified mediation models with child adversity as predictor and only one mediator (distress or neuroticism) and only one outcome variable (reasoning or processing speed).*

|  | B | SE | z | p | 95% CI LB | 95% CI UB |
| --- | --- | --- | --- | --- | --- | --- |
| Model: Childhood adversity → Distress → Reasoning | | | | | | |
| Direct effect |  |  |  |  |  |  |
| Child adversity → Reasoning | -0.045 | 0.005 | -8.740 | 0.000 | -0.055 | -0.035 |
| Indirect effect |  |  |  |  |  |  |
| Child adversity → Distress → Reasoning ^a^ | -0.009 | 0.001 | -9.598 | 0.000 | -0.011 | -0.007 |
| Components |  |  |  |  |  |  |
| Child adversity → Distress | 0.184 | 0.004 | 51.180 | 0.000 | 0.177 | 0.191 |
| Distress → Reasoning | -0.051 | 0.005 | -9.790 | 0.000 | -0.061 | -0.041 |
| Model: Childhood adversity → Distress → Processing speed | | | | | | |
| Direct effect |  |  |  |  |  |  |
| Child adversity → Processing speed | 0.007 | 0.003 | 2.250 | 0.025 | 0.001 | 0.014 |
| Indirect effect |  |  |  |  |  |  |
| Child adversity → Distress → Processing speed ^a^ | 0.006 | 0.001 | 6.816 | 0.000 | 0.004 | 0.008 |
| Components |  |  |  |  |  |  |
| Child adversity → Distress | 0.194 | 0.002 | 78.790 | 0.000 | 0.189 | 0.199 |
| Distress → Processing speed | 0.031 | 0.004 | 6.850 | 0.000 | 0.022 | 0.039 |
| Model: Childhood adversity → Neuroticism → Reasoning | | | | | | |
| Direct effect |  |  |  |  |  |  |
| Child adversity → Reasoning | -0.042 | 0.005 | -7.980 | 0.000 | -0.053 | -0.032 |
| Indirect effect |  |  |  |  |  |  |
| Child adversity → Neuroticism → Reasoning ^a^ | -0.007 | 0.001 | -7.815 | 0.000 | -0.008 | -0.005 |
| Components |  |  |  |  |  |  |
| Child adversity → Neuroticism | 0.155 | 0.004 | 40.780 | 0.000 | 0.148 | 0.163 |
| Neuroticism → Reasoning | -0.043 | 0.005 | -7.980 | 0.000 | -0.054 | -0.033 |
| Model: Childhood adversity → Neuroticism → Processing speed | | | | | | |
| Direct effect |  |  |  |  |  |  |
| Child adversity → Processing speed | 0.009 | 0.003 | 3.330 | 0.001 | 0.004 | 0.015 |
| Indirect effect |  |  |  |  |  |  |
| Child adversity → Neuroticism → Processing speed ^a^ | 0.004 | 0.000 | 9.023 | 0.000 | 0.003 | 0.005 |
| Components |  |  |  |  |  |  |
| Child adversity → neuroticism | 0.158 | 0.003 | 60.570 | 0.000 | 0.153 | 0.164 |
| Neuroticism → Processing speed | 0.026 | 0.003 | 9.140 | 0.000 | 0.020 | 0.031 |

*Note.* Results are adjusted for covariates (sex, age, Townsend deprivation index, years of education). β: Standardized path coefficient, SE: standard error, CI: confidence Interval, LL: lower limit, UL: upper limit. ^a^ partial mediation.

**Table S6.**

*Path analyses using simplified mediation models with child adversity as predictor and only one mediator (distress or neuroticism) and only one outcome variable (reasoning or processing speed).*

| Effect | B | SE | z | p | 95% CI LB | 95% CI UB |
| --- | --- | --- | --- | --- | --- | --- |
| Model: Adulthood adversity → Distress → Reasoning | | | | | | |
| Direct effect |  |  |  |  |  |  |
| Adult adversity → Reasoning | -0.097 | 0.005 | -18.680 | 0.000 | -0.107 | -0.087 |
| Indirect effect |  |  |  |  |  |  |
| Adult adversity → Distress → Reasoning ^a^ | -0.008 | 0.001 | -7.893 | 0.000 | -0.010 | -0.006 |
| Components |  |  |  |  |  |  |
| Adult adversity → Distress | 0.192 | 0.004 | 52.310 | 0.000 | 0.185 | 0.199 |
| Distress → Reasoning | -0.042 | 0.005 | -8.000 | 0.000 | -0.052 | -0.032 |
| Model: Adulthood adversity → Distress → Processing speed | | | | | | |
| Direct effect |  |  |  |  |  |  |
| Adult adversity → Processing speed | 0.032 | 0.004 | 7.560 | 0.000 | 0.024 | 0.040 |
| Indirect effect |  |  |  |  |  |  |
| Adult adversity → Distress → Processing speed ^a^ | 0.005 | 0.001 | 7.237 | 0.000 | 0.004 | 0.006 |
| Components |  |  |  |  |  |  |
| Adult adversity → Distress | 0.191 | 0.003 | 75.640 | 0.000 | 0.186 | 0.196 |
| Distress → Processing speed | 0.026 | 0.004 | 7.280 | 0.000 | 0.019 | 0.033 |
| Model: Adulthood adversity → Neuroticism → Reasoning | | | | | | |
| Direct effect |  |  |  |  |  |  |
| Adult adversity → Reasoning | -0.093 | 0.005 | -17.310 | 0.000 | -0.104 | -0.083 |
| Indirect effect |  |  |  |  |  |  |
| Adult adversity → Neuroticism → Reasoning ^a^ | -0.005 | 0.001 | -6.816 | 0.000 | -0.007 | -0.004 |
| Components |  |  |  |  |  |  |
| Adult adversity → Neuroticism | 0.139 | 0.004 | 35.700 | 0.000 | 0.132 | 0.147 |
| Neuroticism → Reasoning | -0.038 | 0.005 | -6.960 | 0.000 | -0.048 | -0.027 |
| Model: Adulthood adversity → Neuroticism → Processing speed | | | | | | |
| Direct effect |  |  |  |  |  |  |
| Adult adversity → Processing speed | 0.033 | 0.005 | 7.230 | 0.000 | 0.024 | 0.042 |
| Indirect effect |  |  |  |  |  |  |
| Adult adversity → Neuroticism → Processing speed ^a^ | 0.003 | 0.001 | 6.422 | 0.000 | 0.002 | 0.004 |
| Components |  |  |  |  |  |  |
| Adult adversity → Neuroticism | 0.140 | 0.003 | 52.010 | 0.000 | 0.135 | 0.145 |
| Neuroticism → Reasoning | 0.024 | 0.004 | 6.480 | 0.000 | 0.016 | 0.031 |

*Note.* Results are adjusted for covariates (sex, age, Townsend deprivation index, years of education). β: Standardized path coefficient, SE: standard error, CI: confidence Interval, LL: lower limit, UL: upper limit. ^a^ partial mediation.

**Table S7.**

*Path analyses showing the effects of child adversity on observed variables indicative of cognitive performance and mediation by distress and neuroticism*

| Effect | B | SE | z | p | 95% CI LB | 95% CI UB |
| --- | --- | --- | --- | --- | --- | --- |
|  |  |  |  |  |  |  |
| Model: Child adversity → Distress/Neuroticism → Digit span | | | | | | |
| Direct effects |  |  |  |  |  |  |
| Child adversity → Digit span | -0.005 | 0.004 | -1.470 | 0.143 | -0.013 | 0.002 |
| Indirect effects |  |  |  |  |  |  |
| Child adversity → Distress → Digit span ^b^ | -0.004 | 0.001 | -5.243 | 0.000 | -0.005 | -0.002 |
| Child adversity → Neuroticism → Digit span ^b^ | -0.003 | 0.001 | -5.344 | 0.000 | -0.005 | -0.002 |
| Components |  |  |  |  |  |  |
| Child adversity → Distress | 0.178 | 0.003 | 51.350 | 0.000 | 0.172 | 0.185 |
| Child adversity → Neuroticism | 0.151 | 0.003 | 43.600 | 0.000 | 0.145 | 0.158 |
| Distress → Digit span | -0.022 | 0.004 | -5.280 | 0.000 | -0.030 | -0.014 |
| Neuroticism → Digit span | -0.022 | 0.004 | -5.400 | 0.000 | -0.030 | -0.014 |
| Model: Child adversity → Distress/Neuroticism → Fluid intelligence | | | | | | |
| Direct effects |  |  |  |  |  |  |
| Child adversity → Fluid intelligence | -0.025 | 0.003 | -7.240 | 0.000 | -0.031 | -0.018 |
| Indirect effects |  |  |  |  |  |  |
| Child adversity → Distress → Fluid intelligence ^a^ | -0.004 | 0.001 | -6.114 | 0.000 | -0.006 | -0.003 |
| Child adversity → Neuroticism → Fluid intelligence | -0.001 | 0.001 | -1.767 | 0.077 | -0.002 | 0.000 |
| Components |  |  |  |  |  |  |
| Child adversity → Distress | 0.181 | 0.003 | 54.460 | 0.000 | 0.175 | 0.188 |
| Child adversity → Neuroticism | 0.152 | 0.003 | 45.720 | 0.000 | 0.145 | 0.158 |
| Distress → Fluid intelligence | -0.024 | 0.004 | -6.170 | 0.000 | -0.031 | -0.016 |
| Neuroticism → Fluid intelligence | -0.007 | 0.004 | -1.770 | 0.076 | -0.014 | 0.001 |
| Model: Child adversity → Distress/Neuroticism → Symbol matches | | | | | | |
| Direct effects |  |  |  |  |  |  |
| Child adversity → Symbol matches | -0.027 | 0.003 | -8.590 | 0.000 | -0.034 | -0.021 |
| Indirect effects |  |  |  |  |  |  |
| Child adversity → Distress → Symbol matches ^a^ | -0.006 | 0.001 | -8.804 | 0.000 | -0.007 | -0.005 |
| Child adversity → Neuroticism → Symbol matches ^a^ | -0.004 | 0.001 | -6.884 | 0.000 | -0.005 | -0.003 |
| Components |  |  |  |  |  |  |
| Child adversity → Distress | 0.182 | 0.003 | 53.800 | 0.000 | 0.175 | 0.189 |
| Child adversity → Neuroticism | 0.152 | 0.003 | 45.040 | 0.000 | 0.146 | 0.159 |
| Distress → Symbol matches | -0.032 | 0.004 | -8.940 | 0.000 | -0.039 | -0.025 |
| Neuroticism → Symbol matches | -0.025 | 0.004 | -6.980 | 0.000 | -0.032 | -0.018 |
| Model: Child adversity → Distress/Neuroticism → Trial Making Test | | | | | | |
| Direct effects |  |  |  |  |  |  |
| Child adversity → Trial Making Test | 0.005 | 0.001 | 5.040 | 0.000 | 0.003 | 0.007 |
| Indirect effects |  |  |  |  |  |  |
| Child adversity → Distress → Trial Making Test ^a^ | 0.005 | 0.001 | 6.382 | 0.000 | 0.003 | 0.007 |
| Child adversity → Neuroticism → Trial Making Test ^a^ | 0.002 | 0.001 | 3.220 | 0.001 | 0.001 | 0.003 |
| Components |  |  |  |  |  |  |
| Child adversity → Distress | 0.180 | 0.004 | 50.100 | 0.000 | 0.173 | 0.187 |
| Child adversity → Neuroticism | 0.152 | 0.004 | 42.180 | 0.000 | 0.145 | 0.159 |
| Distress → Trial Making Test | 0.008 | 0.001 | 6.440 | 0.000 | 0.005 | 0.010 |
| Neuroticism → Trial Making Test | 0.004 | 0.001 | 3.230 | 0.001 | 0.002 | 0.006 |
| Model: Child adversity → Distress/Neuroticism → Reaction time | | | | | | |
| Direct effects |  |  |  |  |  |  |
| Child adversity → Reaction time | 0.005 | 0.002 | 2.130 | 0.033 | 0.000 | 0.010 |
| Indirect effects |  |  |  |  |  |  |
| Child adversity → Distress → Reaction time ^a^ | 0.004 | 0.001 | 6.444 | 0.000 | 0.003 | 0.005 |
| Child adversity → Neuroticism → Reaction time ^a^ | 0.003 | 0.000 | 5.490 | 0.000 | 0.002 | 0.004 |
| Components |  |  |  |  |  |  |
| Child adversity → Distress | 0.190 | 0.003 | 72.400 | 0.000 | 0.185 | 0.195 |
| Child adversity → Neuroticism | 0.155 | 0.003 | 59.820 | 0.000 | 0.150 | 0.160 |
| Distress → Reaction time | 0.015 | 0.003 | 5.520 | 0.000 | 0.010 | 0.020 |
| Neuroticism → Reaction time | 0.017 | 0.003 | 6.480 | 0.000 | 0.012 | 0.023 |
| Model: Child adversity → Distress/Neuroticism → RT variability | | | | | | |
| Direct effects |  |  |  |  |  |  |
| Child adversity → RT variability | 0.000 | 0.000 | 1.570 | 0.117 | 0.000 | 0.001 |
| Indirect effects |  |  |  |  |  |  |
| Child adversity → Distress → RT variability ^a^ | 0.001 | 0.001 | 2.326 | 0.020 | 0.000 | 0.003 |
| Child adversity → Neuroticism → RT variability | 0.001 | 0.000 | 1.214 | 0.225 | 0.000 | 0.002 |
| Components |  |  |  |  |  |  |
| Child adversity → Distress | 0.190 | 0.003 | 72.400 | 0.000 | 0.185 | 0.195 |
| Child adversity → Neuroticism | 0.155 | 0.003 | 59.820 | 0.000 | 0.150 | 0.160 |
| Distress → RT variability | 0.000 | 0.000 | 2.330 | 0.020 | 0.000 | 0.001 |
| Neuroticism → RT variability | 0.000 | 0.000 | 1.210 | 0.225 | 0.000 | 0.001 |

*Note.* Results are adjusted for covariates (sex, age, Townsend deprivation index, years of education). β: Standardized path coefficient, SE: standard error, CI: confidence Interval, LL: lower limit, UL: upper limit. ^a^ partial mediation, ^b^ complete mediation.

**Table S8.**

*Path analyses showing the effects of adulthood adversity on observed variables indicative of cognitive performance and mediation by distress and neuroticism*

| Effect | B | SE | z | p | 95% CI LB | 95% CI UB |
| --- | --- | --- | --- | --- | --- | --- |
| Model: Adult adversity→ Distress/Neuroticism → Digit span | | | | | | |
| Direct effects |  |  |  |  |  |  |
| Adult adversity → Digit span | -0.026 | 0.004 | -6.810 | 0.000 | -0.033 | -0.018 |
| Indirect effects |  |  |  |  |  |  |
| Adult adversity → Distress → Digit span ^a^ | -0.004 | 0.001 | -4.568 | 0.000 | -0.005 | -0.002 |
| Adult adversity → Neuroticism → Digit span ^a^ | -0.003 | 0.001 | -5.128 | 0.000 | -0.004 | -0.002 |
| Components |  |  |  |  |  |  |
| Adult adversity → Distress | 0.188 | 0.004 | 51.790 | 0.000 | 0.181 | 0.195 |
| Adult adversity → Neuroticism | 0.142 | 0.004 | 38.900 | 0.000 | 0.134 | 0.149 |
| Distress → Digit span | -0.019 | 0.004 | -4.600 | 0.000 | -0.027 | -0.011 |
| Neuroticism → Digit span | -0.021 | 0.004 | -5.190 | 0.000 | -0.030 | -0.013 |
| Model: Adult adversity→ Distress/Neuroticism → Fluid intelligence | | | | | | |
| Direct effects |  |  |  |  |  |  |
| Adult adversity → Fluid intelligence | -0.068 | 0.004 | -19.300 | 0.000 | -0.075 | -0.061 |
| Indirect effects |  |  |  |  |  |  |
| Adult adversity → Distress → Fluid intelligence ^a^ | -0.003 | 0.001 | -4.557 | 0.000 | -0.005 | -0.002 |
| Adult adversity → Neuroticism → Fluid intelligence | -0.001 | 0.001 | -1.128 | 0.259 | -0.002 | 0.000 |
| Components |  |  |  |  |  |  |
| Adult adversity → Distress | 0.188 | 0.003 | 54.120 | 0.000 | 0.181 | 0.194 |
| Adult adversity → Neuroticism | 0.139 | 0.003 | 40.120 | 0.000 | 0.132 | 0.146 |
| Distress → Fluid intelligence | -0.018 | 0.004 | -4.580 | 0.000 | -0.025 | -0.010 |
| Neuroticism → Fluid intelligence | -0.004 | 0.004 | -1.130 | 0.257 | -0.012 | 0.003 |
| Model: Adult adversity→ Distress/Neuroticism → Symbol matches | | | | | | |
| Direct effects |  |  |  |  |  |  |
| Adult adversity → Symbol matches | -0.042 | 0.003 | -12.510 | 0.000 | -0.048 | -0.035 |
| Indirect effects |  |  |  |  |  |  |
| Adult adversity → Distress → Symbol matches ^a^ | -0.006 | 0.001 | -8.371 | 0.000 | -0.007 | -0.004 |
| Adult adversity → Neuroticism → Symbol matches ^a^ | -0.003 | 0.001 | -6.856 | 0.000 | -0.004 | -0.002 |
| Components |  |  |  |  |  |  |
| Adult adversity → Distress | 0.189 | 0.004 | 53.540 | 0.000 | 0.182 | 0.196 |
| Adult adversity → Neuroticism | 0.139 | 0.004 | 39.250 | 0.000 | 0.132 | 0.146 |
| Distress → Symbol matches | -0.031 | 0.004 | -8.490 | 0.000 | -0.038 | -0.024 |
| Neuroticism → Symbol matches | -0.025 | 0.004 | -6.980 | 0.000 | -0.032 | -0.018 |
| Model: Adult adversity→ Distress/Neuroticism → Trial Making Test | | | | | | |
| Direct effects |  |  |  |  |  |  |
| Adult adversity → Trial Making Test | 0.007 | 0.001 | 6.080 | 0.000 | 0.005 | 0.009 |
| Indirect effects |  |  |  |  |  |  |
| Adult adversity → Distress → Trial Making Test ^a^ | 0.005 | 0.001 | 5.941 | 0.000 | 0.003 | 0.007 |
| Adult adversity → Neuroticism → Trial Making Test ^a^ | 0.002 | 0.001 | 3.231 | 0.001 | 0.001 | 0.003 |
| Components |  |  |  |  |  |  |
| Adult adversity → Distress | 0.192 | 0.004 | 51.300 | 0.000 | 0.185 | 0.199 |
| Adult adversity → Neuroticism | 0.138 | 0.004 | 36.780 | 0.000 | 0.131 | 0.146 |
| Distress → Trial Making Test | 0.007 | 0.001 | 6.080 | 0.000 | 0.005 | 0.009 |
| Neuroticism → Trial Making Test | 0.004 | 0.001 | 3.250 | 0.001 | 0.002 | 0.006 |
| Model: Adult adversity→ Distress/Neuroticism → Reaction time | | | | | | |
| Direct effects |  |  |  |  |  |  |
| Adult adversity → Reaction time | 0.025 | 0.002 | 10.100 | 0.000 | 0.020 | 0.030 |
| Indirect effects |  |  |  |  |  |  |
| Adult adversity → Distress → Reaction time ^a^ | 0.003 | 0.001 | 5.317 | 0.000 | 0.002 | 0.004 |
| Adult adversity → Neuroticism → Reaction time ^a^ | 0.002 | 0.000 | 5.105 | 0.000 | 0.001 | 0.003 |
| Components |  |  |  |  |  |  |
| Adult adversity → Distress | 0.188 | 0.003 | 69.580 | 0.000 | 0.183 | 0.194 |
| Adult adversity → Neuroticism | 0.138 | 0.003 | 51.440 | 0.000 | 0.132 | 0.143 |
| Distress → Reaction time | 0.014 | 0.003 | 5.340 | 0.000 | 0.009 | 0.020 |
| Neuroticism → Reaction time | 0.014 | 0.003 | 5.140 | 0.000 | 0.009 | 0.019 |
| Model: Adult adversity→ Distress/Neuroticism → RT variability | | | | | | |
| Direct effects |  |  |  |  |  |  |
| Adult adversity → RT variability | 0.001 | 0.000 | 3.350 | 0.001 | 0.000 | 0.001 |
| Indirect effects |  |  |  |  |  |  |
| Adult adversity → Distress → RT variability | 0.001 | 0.001 | 2.077 | 0.038 | 0.000 | 0.002 |
| Adult adversity → Neuroticism → RT variability | 0.001 | 0.000 | 1.351 | 0.177 | 0.000 | 0.001 |
| Components |  |  |  |  |  |  |
| Adult adversity → Distress | 0.188 | 0.003 | 69.580 | 0.000 | 0.183 | 0.194 |
| Adult adversity → Neuroticism | 0.138 | 0.003 | 51.440 | 0.000 | 0.132 | 0.143 |
| Distress → RT variability | 0.000 | 0.000 | 2.080 | 0.038 | 0.000 | 0.001 |
| Neuroticism → RT variability | 0.000 | 0.000 | 1.350 | 0.176 | 0.000 | 0.001 |

*Note.* Results are adjusted for covariates (sex, age, Townsend deprivation index, years of education). β: Standardized path coefficient, SE: standard error, CI: confidence Interval, LL: lower limit, UL: upper limit. ^a^ partial mediation.
